# Understanding Neuropsychiatric Diseases, Analyzing the Peptide Sharing between Infectious Agents and the Language-Associated NMDA 2A Protein

**DOI:** 10.3389/fpsyt.2016.00060

**Published:** 2016-04-11

**Authors:** Guglielmo Lucchese

**Affiliations:** ^1^Brain and Language Laboratory, Freie Universität Berlin, Berlin, Germany

**Keywords:** neuropsychiatric diseases, language disorders, infectious agents, NMDA 2A, peptide sharing, cross-reactivity

## Abstract

Language disorders and infections may occur together and often concur, to a different extent and via different modalities, in characterizing brain pathologies, such as schizophrenia, autism, epilepsies, bipolar disorders, frontotemporal neurodegeneration, and encephalitis, *inter alia*. The biological mechanism(s) that might channel language dysfunctions and infections into etiological pathways connected to neuropathologic sequelae are unclear. Searching for molecular link(s) between language disorders and infections, the present study explores the language-associated NMDA 2A subunit for peptide sharing with pathogens that have been described in concomitance with neuropsychiatric diseases. It was found that a vast peptide commonality links the human glutamate ionotropic receptor NMDA 2A subunit to infectious agents. Such a link expands to and interfaces with neuropsychiatric disorders in light of the specific allocation of NMDA 2A gene expression in brain areas related to language functions. The data hint at a possible pathologic scenario based on anti-pathogen immune responses cross-reacting with NMDA 2A in the brain.

## Introduction

Neuropsychiatric diseases, such as frontotemporal degeneration, schizophrenia, autism, epilepsies, and bipolar disorders, are, in general, accompanied by language dysfunctions that have been clinically described and tentatively classified into variants and subvariants, such as progressive non-fluent aphasia, logopenic primary progressive aphasia, semantic variant primary progressive aphasia, non-fluent speech out-put, expressive aphasia, loss of word meaning, impaired word comprehension, dysphasic dementia, speech apraxia, fluent speech with anomia, and schizophasia, *inter alia* (1–3). Speech and language disturbance manifestations may be present from the beginning of the neuropsychiatric disease, may be transient and reversibile, or can progress toward full-blown aphasia; or may gradually develop during progressive brain decay such as frontotemporal lobar degeneration. On the whole, the multiplicity of language disorders and their definitions mirrors the complexity of the symptoms of the associated neuropsychiatric disorders (4, 5). At the molecular level, the determinant(s) and mechanism(s) that relate neuropsychiatric diseases to language alteration/disintegration remain poorly understood.

Genes coding for neuropathological proteins, such as chromatin-modifying protein 2b (CHMP2B), protein chromosome 9 open-reading-frame 72 (C9ORF72), granulin (GRN), microtubule-associated protein tau (TAU), and the glutamate receptor ionotropic *N*-methyl-d-aspartate receptor subunit 2A (NMDA 2A), have been intensively investigated as possible genetic markers of neuropsychiatric diseases and/or language disorders (6–8). Nonetheless, the relationship between gene mutations, neuropathologies, and/or language dysfunctions remains obscure. For example, studies on frontotemporal dementia (FTD) and the accompanying language dysfunction antigens showed: the presence of C9ORF72 mutations in only 6.8% of primary progressive aphasia patients (9); low frequency of GRN gene mutations (about 6%) (10–12); lack of a solid association between mutations in the three genes most commonly associated with FTD (C9ORF72, TAU, and GRN) and progressive apraxia of speech and primary progressive aphasia (13); absence of TAU mutations in 25 patients with primary progressive aphasia (14); that 73% of 364 patients with FTD did not have clear evidence of a genetic abnormality (12); and, in addition, that GRN contains a high percentage of non-pathogenic variants and variants of unclear pathogenicity, thus underlining the need of discriminating between genetically normal variants and pathogenic mutations (15).

Among the studies cited above (6–16), a genome-wide association analysis on FTD and its subtypes (16) shifted the scientific attention from gene mutations to an immune context. The study was conducted on 3,526 patients and 7,444 healthy subjects. FTD patients comprehended 308 cases of semantic dementia versus 616 controls, and 269 cases of progressive non-fluent aphasia versus 538 controls. In the end, the main conclusion of the study pointed to a link between FTD and the immune system, thus supporting the idea that immune reactions play an important role in the pathogenesis of neurodegenerative diseases. Moreover, a vast corpus of data has called attention to the possibility that anti-NMDA receptor immunoreactivity may be linked to neuropathologies and cognitive decline. Indeed, anti-NMDA receptor antibodies have been described in encephalitis patients (17), different dementia types (18), and schizophrenia, schizoaffective disorder, mania, bipolar disorder, and major depressive disorder (19).

Finally, clinical and epidemiologic data introduced infections as a third player in the context of neuropsychiatric disorders and anti-NMDA receptor immunoreactivity. An association with infectious agents has been repeatedly suggested for psychoses (20), schizophrenia (21–23), autism (24, 25), aphasia (26–30), and encephalitis, often in concomitance with anti-NMDA receptor antibodies (31–35). The issue is further complicated by the plethora of pathogens that populate the clinical reports on neuroinfections (24, 31–33). Taken together, these studies posit together NMDA receptor, neuropsychiatric diseases that entail language impairment, and a vast array of pathogens into a multifactorial scenario, the molecular connections of which remain intricate, hypothetical, and unclear.

On these premises, the present investigation focused on a preliminary research question: might anti-NMDA receptor antibodies originate from sequences common to the ionotropic glutamate receptor and infectious antigens? As a matter of fact, when pathogens share peptide sequences with host proteins, then an immune response against the pathogen might result in autoimmune cross-reactions against the host proteins with consequent autoimmune damage to the tissue(s) expressing the proteins (36). Therefore, it was reasoned that peptide commonalities, if any, between NMDA receptor and pathogens would have as a consequence that anti-pathogen immune responses might cross-react with the NMDA receptor and, consequently, hit and damage the neural tissues that allocate the glutamate ionotropic receptor. Hence, the kind and the extent of neurological sequelae would depend on the type of immune response, the antibody affinity and avidity, the possibility of concomitant immune phenomena, such as epitope spreading, the spatio-temporal expression of the glutamate ionotropic receptor, and, obviously, the integrity of the blood–brain barrier.

Along this research rationale that had been already applied in analyzing the peptide sharing between influenza A H1N1 hemagglutinin and human axon guidance proteins (23), this study uses as a model NMDA 2A, i.e., a NMDA receptor subunit that has been associated with language (7, 8) searching for peptide sequences in common with pathogens. The results document a vast peptide commonality between the language-associated protein and numerous infectious agents, thus showing the possibility of cross-reactions with the neural receptor subunit following pathogen infections. The data are discussed in light of the specific NMDA 2A expression in brain language areas.

## Methods

Sequence matching analyses were conducted on the human glutamate ionotropic receptor *N*-methyl-d-aspartate receptor subunit 2A (NMDA 2A, 1,464 amino acids) described at http://www.uniprot.org/uniprot/Q12879. NMDA 2A primary sequence was dissected into heptapeptides offset by one residues: i.e., MGRVGYW, GRVGYWT, RVGYWTL, VGYWTLL, etc. Then, each NMDA 2A heptapeptide was analyzed for exact matching against the proteome database as represented by UniProtKB^1^ using the Protein Information Resource perfect batch program^2^ (37). The UniProt Knowledgebase (UniProtKB) is the central hub for the collection of functional information on proteins from known species (580,793 at the time of this study). Matches with proteins from pathogens that have been reported or mentioned in neurological disorders (20–35) were captured and recorded.

Data on NMDA 2A gene expression were generously provided by GENEINVESTIGATOR (38); the data derive from expression mapping across 384 tissues by using the Affimetrix GeneChip^®^ Human Genome U133 Plus 2.0 Array, a most comprehensive whole human genome expression.

## Results

### Peptide Sharing between the Language-Associated NMDA 2A Molecule and Infectious Agents

Table 1 shows a synthesis of the heptapeptide overlap between the NMDA 2A protein and pathogen proteomes. In discussing Table 1 two premises are due. First, given the high number of pathogen species and subspecies that have been described or mentioned in neurodegenerative diseases, the data reported here refer to representative pathogens. Second, it has to be noted that the extent of the heptapeptide sharing reported in Table 1 is mathematically unexpected. Indeed, assuming that the 20 amino acids that form proteins occur with the same frequency and without considering protein length, the probability of a sequence of 7 amino acids occurring at random in two proteins is equal to 20^−7^, i.e., there is 1 probability out 1,280,000,000 of sharing a heptapeptide. In light of this mathematical notion, the peptide sharing shown in Table 1 is impressive and appears even more striking at level of *Echinococcus granulosus*, *Echinococcus multilocularis*, and *Schistosoma mansoni*, which share a same 14-mer peptide (FLASYTANLAAFMI) with the NMDA 2A protein against a mathematical expectation practically equal to 0 (that is, 20^−14^).

**Table 1:** **Peptide sharing between infectious agents and the human NMDA 2A protein**.

Infectious agent	Heptapeptide(s) shared with NMDA 2A^a^
**Viruses**	
Hepatitis C virus	LLWGLVF
Human herpes simplex virus 2	PAPSAAA
Human herpes virus 8	SRGIYSC
Human immunodeficiency virus 1	DGEMEEL; DIDPLTE; INNSTNE; KINNSTN; KTTVDNS; SKIMVSV; STESKAN; TESKANS; WKKSVDS
Human rotavirus A	SKRSKSL
Influenza A virus	IVLNKDR; VLNKDRE
West Nile virus	GILTTAA
**Bacteria**	
*Acidovorax* species	LVLPALL; PAPSAAA; APSAAAE; QAAGLPL; GIGILTT; IGILTTA
*Bartonella* species	AAAMALS; EARVRDG; GLVFNNS; GMIGEV; KGVEDAL; LEPFSAS; LLVLPAL; RDGIGIL; RELDLSR; SQSNMLK; VRDGIGI
*Bordetella pertussis*	GLVFNNS; SDRPGLL
*Borrelia burgdorferi*	FVPILGI; LEKFSYI
*Clostridium difficile*	AAMALSL; APSAAAE; EDALVSL; FAVIFLA; IEEKKKS; ILTTAAS; ISLKDRE; KDEAVLI; KDNLNNY; KNHKTKD; LFSVPSS; LQLQKNK; LTINEER; MVSDKGN; NDQYKLY; NVTLPEN; SLEARVR; TINEERS; TSFEDAK; TTVDNSF; VITLDTS; VMLGHSH; YKAGRDE; YKKMPSI
*Clostridium bolteae*	ETGISVM; GLSDKKF; SQSNMLK; VSDKGNL
*Clostridium* sp. (environmental samples)	AAAMALS; AAGLPLD; AAMALSL; AKNISSM; ALLVWRG; ANLAAFM; ARSLGLT; ASIQQQA; ASMIMAD; AVAQMLD; DDQRLVI; DDWDYSL; DILKKLS; DISETSN; DQYKLYS; DRERLLE; DVNVVAL; EEGLSNN; EEKKKSP; ELDLSRP; ENVTLPE; ERTYLKT; EVAVSTE; FGASIQQ; FISSHTF; FSYIPEA; FVIVEDI; GASIQQQ; GDGEMEE; GEVVYQR; GKLDAFI; GLEDSKR; GLLFSIS; GLVFNNS; IDILKKL; IEEKKKS; ILSEARS; INNSTNE; IPKEFPS; ISSMSNM; ISVSYDD; IVPSLVS; IVSAIAV; KDRERLL; KGTTSKI; KINNSTN; KKKSPDF; KKLSRTV; KLDAFIY; KLLRSAK; KLSGKKS; KNKLRIS; KTTVDNS; LAAAMAL; LFPQGLE; LISVSYD; LLIVSAI; LLVLPAL; LSFTEEG; LTINEER; LVSGNTE; LWKKSVD; LYDIDED; MALSLIT; MLAAAMA; NDQYKLY; NGTVSPS; NIAVMLG; NIRNNYP; NLYDIDE; NVTLPEN; PFVIVED; PSLVSGN; PSSKLSG; QAAGLPL; QMLQETG; RDKIYTI; RELDLSR; RNLAKGK; SDISETS; SGLISVS; SISRGIY; SKSLLPD; SLFPQGL; SLPSQAV; SSKLSGK; SYDNIVD; SYLRSSL; TELIPKE; TFVPILG; TIDGEKE; VASKYPK; VENVTLP; VEVAVST; VFNNSVP; VIVLNKD; VSGNTEL; VTLPENV; VVALLMN; YDIDEDQ; YLVTNGK; YTIDGEK
*Clostridium tetani*	DQYKLYS; FFWIVPSL; FYGSLFS; SLVTTIF
*Corynebacterium diphtheriae*	HNEEGLS; LVLPALL
*Escherichia coli*	AAAMALS; ALSLITF; ALVSLKT; AVIFLAS; DILKKLS; DRERLLE; ESKANSR; GISVMVS; GLVFNNS; KKSVDSI; LASYTAN; LKKLSRT; LKTGKLD; LPSQAVN; LVTIGSG; NPATGEQ; NVTLPEN; PATGEQV; PVQNPKG; QIDLALL; RAADFIQ; RPSRSIS; RQIDLAL; RSEVVDF; SGKKSSL; SKLSGKK; SLFSVPS; SLKTGKL; SNPNTVE; SYTANLA; VWAFFAV; YTANLAA
*Haemophilus influenzae*	DKDPTST; GIGILTT; IGILTTA; KDNLNNY; LVFGDDT; NPATGEQ; SGLISVS
*Helicobacter pylori*	AVIFLAS; LEPFSAS; LIVSAIA; QAAGLPL; TLNESNP; TNGKHGK
*Klebsiella oxytoca*	AAMALSL; ENHTLSL; ETLWLTG; FKRSVAS; KKIHSSV; LDFISSH; LIVSAIA; LVLPALL; LVTIGSG; NMNSSRM; NVITLDT; QIDLALL; QQATVML; TLPENVD; VEDALVS; VTERELR
*Klebsiella pneumoniae*	AAAMALS; APSAAAE; DRERLLE; EARSLGL; FKRSVAS; FSLVTTI; IRNNYPY; KKIHSSV; LDFISSH; LIVSAIA; LLPDHTS; LVLPALL; NPATGEQ; NVITLDT; QIDLALL; RAADFIQ; RSEVVDF; SGLISVS; SNPNTVE; TLPENVD; VTLPENV; VVDFSVP; VWAFFAV
*Mycobacterium tuberculosis*	AFIYDAA; DRPGLLF; GPAPSAA; ILSEARS; LVLPALL; MLAAAMA; PAPSAAA; RVRDGIG; SLRSTAS; TVPNGST; VLPALLV; VSTESKA
*Mycoplasma pneumoniae*	DILKKLS; GILTTAA; LLWGLVF; LVLPALL; PNTVEVA; SLGLTGY; VIVLNKD
*Neisseria meningitidis*	ARSLGLT; GNPATGE; TGNPATG
*Salmonella enterica*	GILTTAA
*Salmonella typhimurium*	APSAAAE; AVIFLAS; IVTLEEA; PAPSAAA; PLTLNES
*Staphylococcus aureus*	DILKKLS; DNHLSIV; IDILKKL; KKIHSSV; KLRISRQ; KRSKSLL; LKKIHSS; LSLITFI; NDSYLRS; SKRSKSL; TGLSDKK; TGNPATG; TLLVLPA; VALLMNR
*Staphylococcus haemolyticus*	LSLITFI; QRDEATA
*Streptococcus agalactiae*	EEGLSNN; FATTGYG; GGASMIM; GILTTAA; IGILTTA; ILTTAAS; KRAADFI; LWLTGIC; NIAVMLG; RAADFIQ; TLWLTGI; TSFEDAK; VLPALLV
*Streptococcus mutans*	ILTTAAS; IVSAIAV; IQEEFVD
*Streptococcus pneumoniae*	EELETLW; GILTTAA; GKDLSFT; GKKVNNV; ILKKLSR; ILTTAAS; LAKGKAP; LKKIHSS; LLVLPAL; SVASKYP; TLPENVD; VITLDTS; VTGLSDK
*Streptococcus pyogenes*	ILTTAAS; KRAADFIQ; LSLITFI; QRAVMAV; TSFEDAKT; VLPALLV
*Streptomyces griseus*	AAGLPLD; FIQRGSL; GPAPSAA; LLVLPAL; LSEARSL; PAPSAAA; PEQAAGL; QHPLTLN; QKGVEDA; RDDQRLV; RLVVIVL; VLPALLV
*Streptomyces* sp.	ATGEQVY; AVLILSE; EQAAGLP; FVANRQK; PAPSAAA; VEVAVST; VTLPENV
**Protozoans**	
*Plasmodium falciparum*	ASSMLEK; GIYSCIH; HIEEKKK; IDILKKLS; KTKDNFK; LLEGNFY; RSAKNIS; SRGIYSC; STNEGMN; SVPSSKL
*Toxoplasma gondii*	AGLPLDV; CSEVERT; DFSVPFV; EARSLGL; ERELRTL; ETGNPAT; GNPATGE; GSPWKRQ; IGILTTA; KGVEDAL; LKKLSRT; LLVLPALL; LRSSLRS; LTTAASS; MLAAAMA; NGTVSPS; PAPSAAA; PLHNEEGL; PSAFLEP; PSLVSGN; PYQDPSE; RERLLEG; RSKSLLP; SPSAFLE; TVSPSAF; VPNGSTE; VPSLVSG; YKHSLPS
*Trypanosoma cruzi*	ALLQFVG; EMEELET; GILTTAAS; KKIHSSV; NPNTVEV; PAPSAAA; RLLEGNF; SLFSVPS; SSMSNMN; TVPNGSTE; VLPALLV
**Fungi**	
*Coccidioides immitis*	AAAMALS; AHSDISE; EDIDPLT; KEPGFHL; LAAAMAL; LPSQAVN; LRSSLRS; LTTAASS; PSIESDV; RELDLSR; RPSRSIS; RSKSLLP; SLVTTIF; TLSLRHA; VLILSEA; VPSLVSG; VTLPENV
*Cryptococcus neoformans*	AMALSLI; ANSRPRQ; ELDLSRP; GKLDAFI; KEFPSGL; MLKLLRS; NLAAFMI; PAPSAAA; PHSETSE; PSLVSGN; RFCFTGV; SPHSETS; TKSSSPR
**Cestodes**	
*Echinococcus granulosus*	FLASYTANLAAFMI^a^
*Echinococcus multilocularis*	FLASYTANLAAFMI^a^
**Trematodes**	
*Schistosoma mansoni*	FLASYTANLAAFMI^a^

*^a^Only the common 14-mer peptide is reported in Table 1 for examined cestode and trematode organisms*.

Looking deeper in Table 1, it can be seen that: among viruses, human immunodeficiency virus 1 has a remarkable level of heptapeptide sharing with the NMDA 2A molecule, for a total of nine matches; in bacterial and protozoan groups, clostridial species and *Toxoplasma gondii* show an impressive hexapeptide overlap to the glutamate ionotropic receptor; heptapeptides from the fungi *Coccidioides immitis* and *Cryptococcus neoformans* are widely represented in NMDA 2A, and cestodes and trematodes, of which only the common 14-mer peptide is reported in Table 1, have an intensive heptapeptide overlap to the human receptor (not shown).

In the end, Table 1 shows that numerous infectious agents share an unexpected high number of heptapeptide sequences with NMDA 2A, thus supporting the possibility of immune cross-reactions between pathogens and NMDA 2A. Examples of potential cross-reactivity are represented by two heptapeptides, MVSDKGN and VSDKGNL, which are shared by NMDA 2A with *Clostridium difficile* and *Clostridium bolteae*, respectively (Table 1). The two heptapeptides are also present in a NMDA 2A-derived epitope MVSDKGNLMY (39). Hence, it is logical to presume that an immune reaction following *C. difficile* infection may occur at level of MVSDKGN and cross-react with the NMDA 2A molecule too, thus possibly contributing to neurological disturbances in the elderly, by being *C. difficile* infection a burden associated with aging (40). In other words, such a cross-reaction might represent a possible link to the neurodegenerative diseases that canonically characterize elderly subjects. Likewise, a cross-reaction against the NMDA 2A VSDKGNL peptide following *C. bolteae* infection might contribute to autistic disorders. Indeed, since 1998, Bolte (41) suggested a role of clostridial infections in autism. Accordingly, *C. bolteae* appears to characterize the gastrointestinal microbiome in children with autism spectrum disorder (42), so to be dubbed as an “autism-associated bacterium” (25). In this regard, the wide heptapeptide platform shared between NMDA 2A and *Clostridium* sp. assumes a special relevance. In synthesis, from a logical point of view, the peptide sharing illustrated in Table 1 suggests the possibility of immune cross-reactivity as a link between the NMDA 2A and infections. Actually, by considering that a minimal immune determinant may be represented by a pentapeptide (43), the number of potential cross-reactions that can derive from the peptide sharing reported in Table 1 is higher as revealed by IEDB analysis (data not shown).

### Mapping NMDA 2A Gene Expression in the Brain

**Figure 1:**
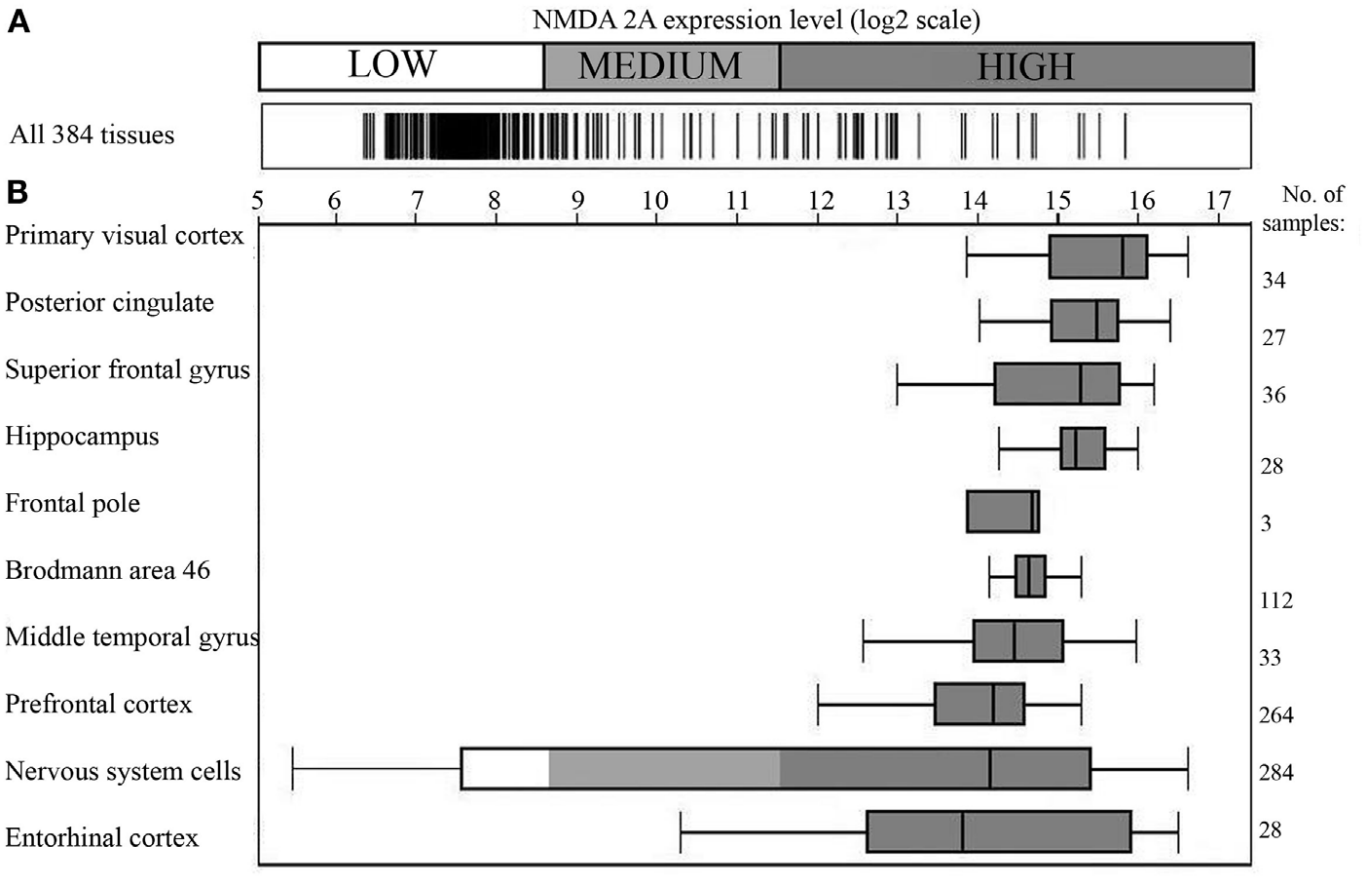
**NMDA 2A gene expression in the human brain**. **(A)** Expression of NMDA 2A across 384 human tissues; **(B)** NMDA 2A expression level in the top ten tissues (ranked on *y* axis on the left). Number of analyzed samples for each tissue on the right. Data obtained using Affimetrix GeneChip^®^Human Genome U133 Plus 2.0 Array; figure modified from the original drawing; data and figure were generously provided by GENEINVESTIGATOR (http://genevisible.com) (38).

As a successive step, correlations with neuropsychiatric alterations were searched by analyzing NMDA 2A gene expression. Figure 1 shows that using microarray technology, a powerful tool for investigating differential gene expression (44), a comparative quantification of NMDA 2A gene expression across human tissues can be obtained (38).

Specifically, Figure 1A shows the gene expression profile of NMDA 2A across 384 human tissues, with the brain presenting the highest expression level (Figure 1B). The top NMDA 2A-expressing brain areas correspond, in the order, to: primary visual cortex, posterior cingulate, superior frontal gyrus, hippocampus, frontal pole, Brodmann area 46, middle temporal gyrus, prefrontal cortex, and entorhinal cortex. In essence, Figure 1B is the graphical representation of the brain NMDA 2A signature profile.

In light of Figure 1, it appears logical to presume that anti-pathogen immune responses cross-reacting with NMDA 2A might have the brain as a predestined “battlefield” and the language-associated areas as preferred sites of cross-reactivity, with consequential deterioration of speech and language. Indeed, primary visual cortex is associated, when lesioned, with alexia without agraphia (45); middle temporal gyrus or Brodmann area 21 is key in processing of text and speech as well as in processing complex sounds (46); hippocampus is involved in the integration of multiple sources of information implicated in language processing (47); posterior cingulate (Brodmann areas 23 and 31) plays a role in semantic retrieval and processing (48); superior frontal gyrus, frontal pole (Brodmann area 10), and the dorsolateral prefrontal cortex (DLPFC) are roughly equivalent to Brodmann areas 9 and 46 and appear to participate in complex language processes such as syntactic processing, metaphor comprehension, generating sentences, and so forth (49); and alterations of entorhinal cortex, a portion of the temporal lobe that channels inputs from neocortex to the hippocampus, are accompanied by semantic dementia (50).

## Discussion

Neurodegenerative disorders may be characterized by manifestations more or less severe, ranging from cognitive decline to brain atrophy (4, 5). A rule almost constant of brain disorders – in autism and schizophrenia as well as in aging and in various forms of frontotemporal neurodegeneration – is represented by the presence of a progressive dissolution of language and speech (1–3). Although numerous factors – from genetic mutations to infections and NMDA immunoreactivity – have been studied in relationship to neurodegenerative processes, clear-cut molecular determinants and mechanisms remain to be established. Likewise, the links between neurodegenerative diseases and language disturbances are unknown.

Given the new attention called to a possible immune context (16) and the re-emergence of neuroinfectiology (51), this study aimed at analyzing the connection between infectious pathogens and anti-NMDA receptor antibodies. The language-associated NMDA 2A subunit (7, 8) was used as a molecular model, and data were obtained that support the possibility of anti-NMDA 2A cross-reactions triggered by infectious pathogens (Table 1) and allocated in the brain (Figure 1).

Indeed, Table 1 and Figure 1 seem to indicate that the “what, how, and where” of neuropsychiatric alterations and associated language deterioration at least in part might reside in: the peptide sharing between pathogens and the language-associated protein NMDA 2A; the consequent potential immune cross-reactivity between pathogens and NMDA 2A; and the high level of NMDA 2A expression in brain areas hosting cognitive, behavioral, and language functions.

In synthesis, the present study supports the hypothesis that infections might play a key role in neuropathology through the language-associated protein NMDA 2A, and invite to expand research on immune cross-reactivity between pathogens and brain antigens. Of special relevance, such a research platform might open the way to preventive and therapeutic protocols to alleviate the devastating burden of neurodegenerative disorders. Of not less importance, the data appear to provide a molecular platform to correlate the heterogeneity of mental disorders and the multitude of viral and bacterial CNS diseases.

## Author Contributions

GL designed the study, interpreted the data, developed the conceptual hypothesis, and wrote the manuscript.

## Conflict of Interest Statement

The author declares that the research was conducted in the absence of any commercial or financial relationships that could be construed as a potential conflict of interest.

## Footnotes

^1^http://www.uniprot.org

^2^http://research.bioinformatics.udel.edu/peptidematch
